# PHYSICAL ACTIVITY AND DEMENTIA PREVENTION: THE ROLE OF HEALTH COGNITIONS AMONG 50+ HEALTHY INDIVIDUALS IN ISRAEL

**DOI:** 10.1093/geroni/igad104.0080

**Published:** 2023-12-21

**Authors:** Offer Edelstein

**Affiliations:** Ben-Gurion University of the Negev, Beer-Sheva, HaDarom, Israel

## Abstract

**Background:**

Although dementia cannot be cured, it can be prevented by adopting various health behaviors, including regular physical activity. The current study sought to i) estimate the participation of Israel-born healthy individuals aged 50 years or older in regular physical activity; ii) assess the associations linking Health Belief Model variables (Rosenstock, 1966, 1974) and engagement in regular physical activity.

**Method:**

This cross-sectional study was conducted in 2021-2022, using online convenience sampling. The study included 328 Israel-born participants aged 50 years or older. Physical activity was assessed by asking whether participants regularly engaged in physical activity (type of activities/times per week/minutes). Cognitive perceptions were assessed using the Motivation to Change Lifestyle and Health Behaviors for Dementia Risk Reduction questionnaire (Kim et al., 2014).

**Results:**

The average weekly minutes of physical activity was 165.62 (S.D. 176.17), whereas only 43.6% performed 150 minutes of physical activity weekly. A multivariate linear regression indicated that among all of the model’s variables, perceived severity (β=-.204, p<.001), cues to action (β=.134, p<.001), feminine gender (β=-.155, p<.01), and low income (β=-.113,p<.05) emerged as significant predictors of weekly minutes of physical activity. The model explained 14.2% of the variance in the performance of weekly minutes of physical activity [F(7,320)=12.22, p<.001].

**Conclusions:**

The current research underlines the role of health cognitions (perceived severity, barriers, and cues for action) regarding engagement in physical activity. The results of the current study might serve as a basis for intervention programs among various target populations.

